# Pleistocene Aridification Cycles Shaped the Contemporary Genetic Architecture of Southern African Baboons

**DOI:** 10.1371/journal.pone.0123207

**Published:** 2015-05-13

**Authors:** Riashna Sithaldeen, Rebecca Rogers Ackermann, Jacqueline M. Bishop

**Affiliations:** 1 Department of Archaeology, University of Cape Town, Private Bag X3, Rondebosch 7701, South Africa; 2 Department of Biological Sciences, University of Cape Town, Private Bag X3, Rondebosch 7701, South Africa; University of Colorado, UNITED STATES

## Abstract

Plio-Pleistocene environmental change influenced the evolutionary history of many animal lineages in Africa, highlighting key roles for both climate and tectonics in the evolution of Africa’s faunal diversity. Here, we explore diversification in the southern African chacma baboon *Papio ursinus sensu lato* and reveal a dominant role for increasingly arid landscapes during past glacial cycles in shaping contemporary genetic structure. Recent work on baboons (*Papio* spp.) supports complex lineage structuring with a dominant pulse of diversification occurring 1-2Ma, and yet the link to palaeoenvironmental change remains largely untested. Phylogeographic reconstruction based on mitochondrial DNA sequence data supports a scenario where chacma baboon populations were likely restricted to refugia during periods of regional cooling and drying through the Late Pleistocene. The two lineages of chacma baboon, *ursinus* and *griseipes*, are strongly geographically structured, and demographic reconstruction together with spatial analysis of genetic variation point to possible climate-driven isolating events where baboons may have retreated to more optimum conditions during cooler, drier periods. Our analysis highlights a period of continuous population growth beginning in the Middle to Late Pleistocene in both the *ursinus* and the PG2 *griseipes* lineages. All three clades identified in the study then enter a state of declining population size (Ne*f*) through to the Holocene; this is particularly marked in the last 20,000 years, most likely coincident with the Last Glacial Maximum. The pattern recovered here conforms to expectations based on the dynamic regional climate trends in southern Africa through the Pleistocene and provides further support for complex patterns of diversification in the region’s biodiversity.

## Introduction

Large-scale environmental change during the Plio-Pleistocene influenced the evolutionary history of many animal lineages in Africa. This is true for many birds e.g. the *Nectarinia*

*olivacea*/*obscura* complex [1] and the forest robins, family Muscicapidae [2], Africa’s herpetofauna including chameleons e.g. the *Bradypodion* complex [3] and snakes e.g. *Bitis arietans* [4] and a large number of mammalian lineages including bovids e.g. *Alcelaphus buselaphus* [5], primates e.g. *Gorilla gorilla* [6]; rodents e.g.the *Heliophobius* argenteocinereus *complex* [7], afrotheria e.g. *Elephantulus edwardii* [8]; bats e.g. the *Rhinolophus hildebrandtii* complex [9] and *Rhinolophus darlingi* complex [10], and suids e.g. *Phacochoerus africanus* [11]. These studies provide compelling evidence for the key roles of both climate and tectonics in the evolution of Africa’s vast faunal diversity. In addition to lineage diversification, environmental change has also driven lineage extinction as evidenced through large-scale shifts in species assemblages (“species turnover” [12]) recorded in the fossil record of Africa [13]. As one example, the expansion of open habitats in East Africa, a result of global cooling and aridification [14,15], facilitated increased diversity of grazing mammals at ~1.8 Ma [16] and similar events may have driven species change in African hominins [17–19]. Understanding the role of climatic and landscape changes in shaping species history is clearly integral to understanding the evolution of Africa’s biodiversity [20]. To achieve this, phylogeographic and phylogenetic methods have emerged as an informative means to reconstruct how species responded to both broad and fine-scale climatic and geomorphic processes across the continent [20–22]. In southern Africa, palaeoenvironmental and biogeographic reconstruction during the Plio-Pleistocene are subjects of growing interest particularly in light of the importance of this region to the evolution of our early human ancestors (e.g. [18,23–25]). Reconstructions are largely dependent on local terrestrial proxy records together with information from distant marine and glacial cores. To date, the picture remains patchy relative to other regions of the world [16,26], with the majority of regional studies focussing on the late Pleistocene and Holocene, i.e. over the last 20–30 thousand years [27–29]. Studies indicate that the southern African region experienced repeated episodes of cooling and drying with the expansion of high latitude ice sheets [14,30], resulting in cycles of intense aridification [31], and patterns of genetic structuring that suggest regional glacial refugia in southern Africa have been recovered in a number of species (e.g. [32]).

Recent phylogenetic reconstructions in the dominant baboon genus of Africa, *Papio*, reveal complex lineage structuring with a dominant pulse of diversification in the genus occurring 1-2Ma [33–35]. First appearance of modern *Papio* in the fossil record is at least 2.5Ma [36]. Thereafter the genus exhibits widespread ecological persistence across wide areas of Africa and currently occupies much of the region south and east of the Sahara, including the Arabian Peninsula [37–39]. At least six species of *Papio* are currently recognized, all of which differ markedly in morphology and behavior, which corresponds to their respective genetic distinctiveness [34,38,40]. Of these, the southern African chacma baboon *Papio ursinus spp*. was the first to diverge from an ancestral Papionin (*ca*. 1.8-2Ma; [33–35]) and likely represents the lineage from which all other baboon species evolved during the Plio-Pleistocene [41]. Numerous authors propose that diversity across Africa’s baboons may have been shaped by climatic fluctuations and environmental cycling in the Pleistocene [33,42,43], though this remains to be tested (but see [35]).

**Fig 1:**
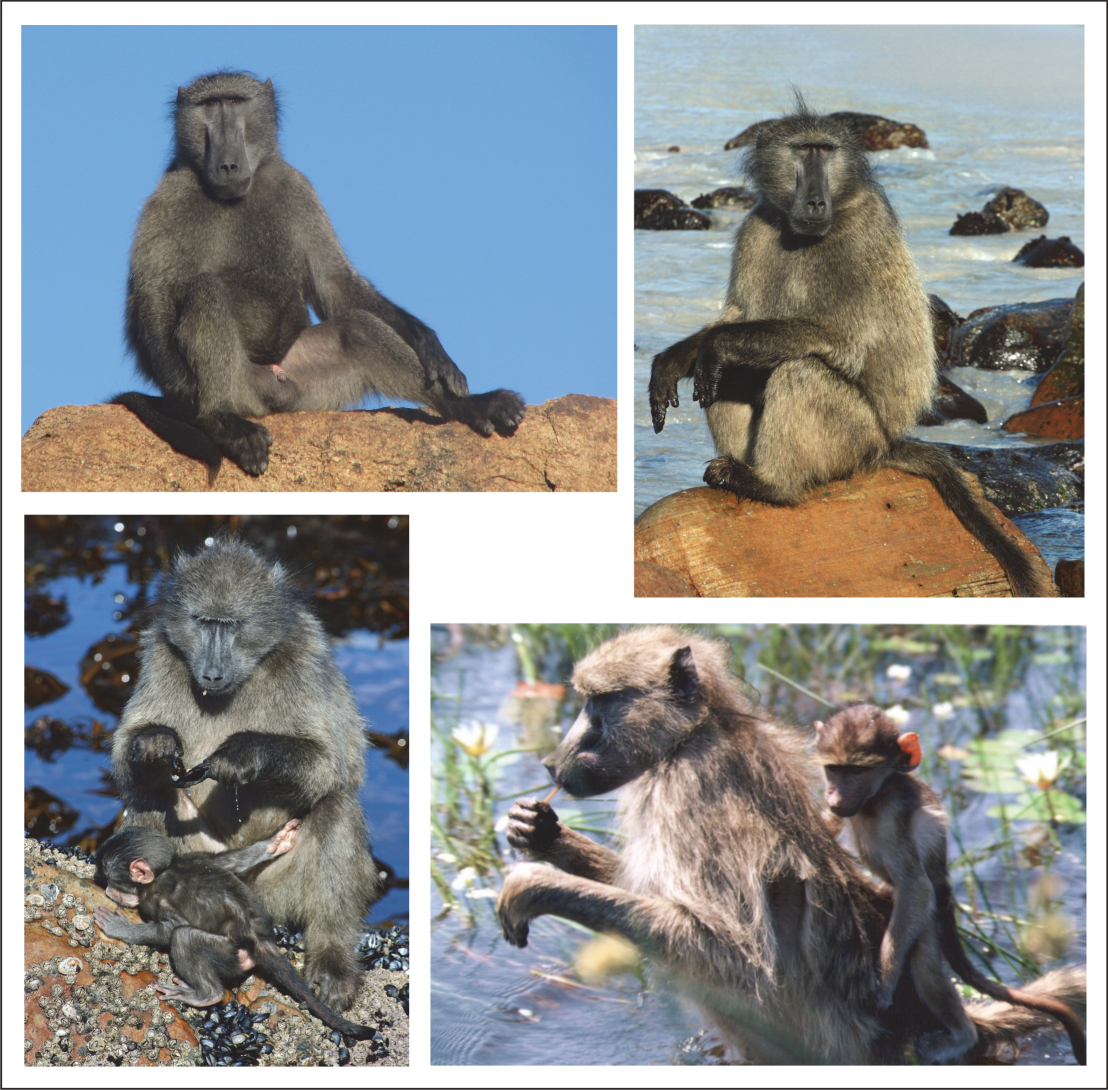
Chacma baboons photographed across the sampling range of this study. Top left: Adult *ursinus* male, Augrabies National Park, South Africa (Photo. R. Sithaldeen). Top right: Adult *ursinus* male, Cape Point, South Africa (Photo. M. Lewis). Bottom left: Adult *ursinus* female and infant, Cape Point, South Africa (Photo. M. Lewis). Bottom right: Adult *griseipes* female and infant, Okavango Delta, South Africa (Photo. R. Ackermann).

Here we use a large mitochondrial dataset sampled across the broad southern African range of chacma baboons *sensu lato* to evaluate the evolutionary history of this charismatic primate (Fig 1); we interpret our findings in light of current models of Pleistocene environmental change in the region. Chacmas are distributed across a wide range of southern African biomes including desert, wetland, savannah, forest and the montane heathlands of the Cape fynbos. In light of recent molecular evidence that has recovered considerable phylogenetic structuring in this taxon, we test for evidence of a link between regional climate change and genetic structure during two established time periods of diversification within chacmas. First we test for evidence of lineage isolation via possible contraction into refugia in response to increasing aridity around 1.7Ma. It is clear that chacma baboons diverged into two distinct mitochondrial clades that fall broadly within the distributions of *Papio ursinus ursinus* and *P*. *ursinus griseipes* [44] (from here forward referred to as the PU clade and PG clade, respectively); this took place 1.9–1.6Ma [33], coincident with one of a number of major shifts in regional aridity [14,31,45]. Second, we explore the role of subsequent glacial cycles in shaping contemporary genetic diversity in PU and PG following their divergence. Our results recover different evolutionary histories for these lineages and highlight the formative role of the Kalahari Desert in shaping the genetic architecture and evolutionary trajectories of these taxa.

## Methods

### Ethics statement

Permission to collect non-invasive faecal samples was obtained from Cape Nature Conservation (Permit #001-201-00004), Ministry of Environment and Tourism Namibia (993/2005) and with assistance from SANParks. Ethical clearance for this study was obtained from the University of Cape Town Science Faculty Animal Ethics Committee (AEC #2006/v2/RS). None of the authors interacted with the animals. There was no work carried out on the animals, and all the sampling was performed in the field.

### Sample collection and DNA sequencing

**Fig 2:**
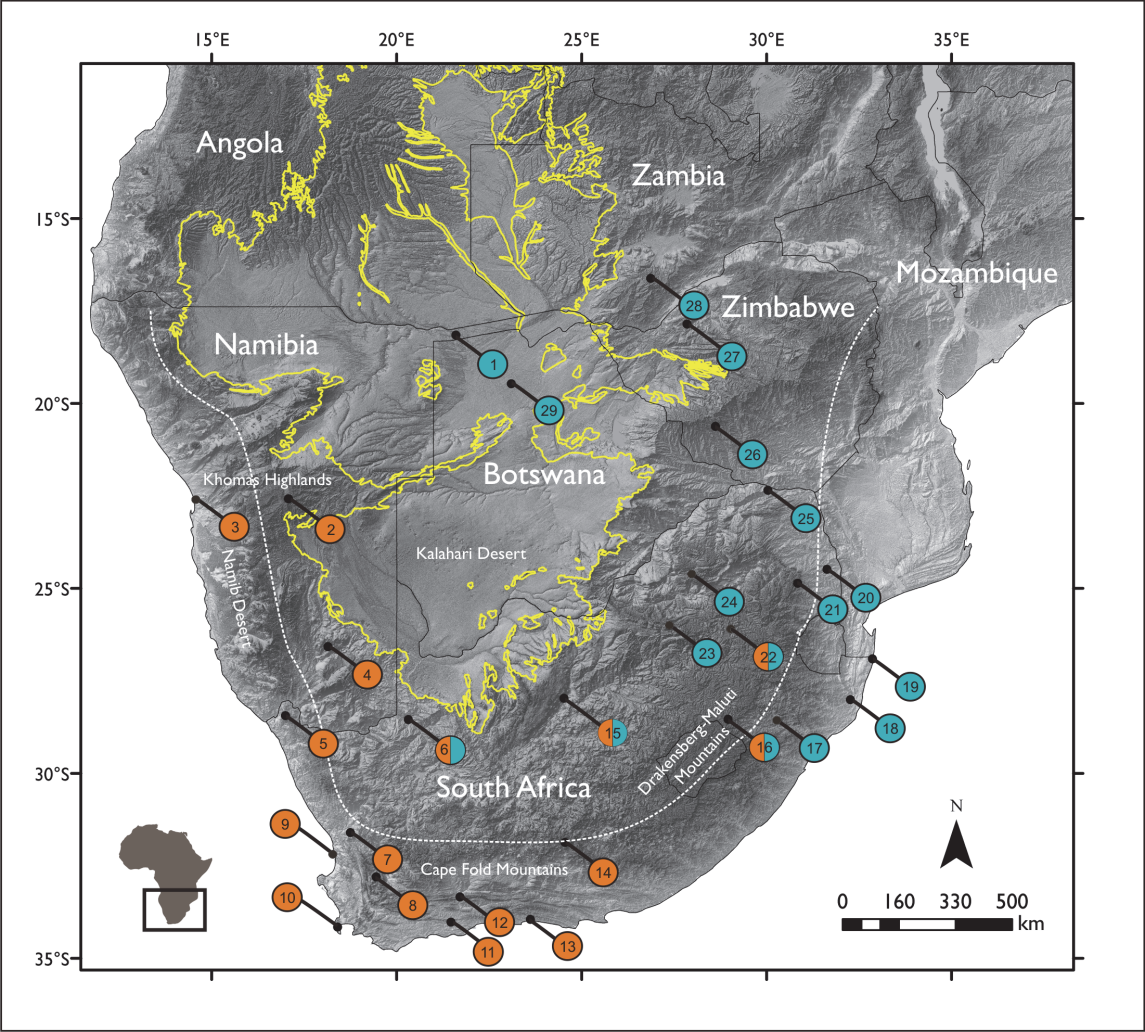
Map of sampling localities across southern Africa. Circles indicate the sampling localities used in this study. Orange circles indicate the *ursinus* mitochondrial lineage, while blue circles indicate the *griseipes* mitochondrial lineage, following [33,43]. Areas where both lineages were sampled are indicated with both colours. The extent of the Kalahari sand geologic formation is denoted by the yellow line. The Great Escarpment of southern Africa is denoted by the white dashed line. The escarpment edges the central southern African plateau and is a major geological formation in Africa. Digital elevation model (DEM) created with data from the NASA Shuttle Radar Topography Mission; all data are available from public domain sources. Sample details, collection site co-ordinates and clade associations are reported in S1 Table. Numbers on the map follow S1 Table.

Fresh faecal material was collected from free-living baboons from 29 localities across southern Africa (Fig 2). Samples were collected directly into 96% ethanol and stored at -20°C. Total DNA was extracted using a QIAamp DNA Stool Kit (Qiagen) following the manufacturer’s instructions and stored at 4°C. High quality DNA was obtained for 135 individuals. Sample details are reported in S1 Table.

We amplified partial sequences for two mitochondrial markers; a 520 base pair (bp) fragment of the D-loop together with the ‘Brown’ region, an 896 bp section comprising 457 bp of the 3’ end of the ND4 gene, tRNA^His^, tRNA^Ser^ and tRNA^Leu^, and 239 bp of the 5’ end of the ND5 gene [46] shown to be phylogenetically informative in baboons [33,47]. Primer sequences and PCR details for amplification of the Brown region are reported in Sithaldeen et al. [33]. The Brown region data analysed here were previously reported in [33] and used in reconstructing the phylogenetic placement of chacma baboons; in this study we use the data set in a different set of analyses, together with D-loop sequences sampled at a finer spatial scale. To sequence the D-loop we used *Papio*-specific PCR primers: H12652 5’AATGTTTGGGTCTGAGTTTATATATCA 3’ from Winney et al.[48] and L11574 5’CTATCCCTATGAGGGATAATTATAAC 3’ designed in this study. PCR reactions for the D-loop were performed in a total volume of 25ul containing 25–50ng of genomic DNA, 1X reaction buffer, 2.5mM MgCl2, 0.2mM dNTPs, 0.5pmol/ul primers, and 1U Taq polymerase (GoTaq, Promega) using an annealing temperature of 54°C. PCRs were performed on a GeneAmp 9700 thermocycler (Applied Biosystems) and sequenced in both directions using BigDye 3.1 chemistry on an ABI 3730 (Applied Biosystems) capillary sequencer. DNA sequences were edited in BIOEDIT v5.0.9 [49] and aligned using CLUSTAL X [50]. The identity of all sequences was checked using the BLASTn search in the NCBI nucleotide database on GenBank and the presence of nuclear copies of mitochondrial genes (numts) was assessed. Numts are a common source of error in studies employing mitochondrial markers [51,52] and have been reported in a number of primates [53–56]. To check for the presence of numts in both our Brown and D-loop region data sets we compared all sequences generated in this study to reference *P*. *ursinus* (AY212105) and *P*. *hamadryas* (AY247447) sequences isolated and sequenced from purified mitochondrial DNA, following Wildman et al. [57]. We also performed replicate PCR and sequencing reactions for comparison in a number of individuals. All sequences analysed aligned to our reference genome and there was no evidence of truncated or chimeric sequences or nonsense mutations in the coding sequences of the Brown region. The final sequence alignments represented 63 unique D-loop sequences, and 34 Brown region sequences. GenBank Accession numbers are JN116728-JN116820 and FJ531498-FJ531532 for the D-loop and Brown region datasets, respectively.

### Testing for genetic isolation using the coalescent

We explored the process of divergence between chacma lineages using the coalescent framework in the program Isolation with Migration (IM) [58]. IM jointly estimates demographic parameters to obtain posterior probability distributions from unlinked molecular markers, revealing the dynamics of populations during early stages of differentiation [59]. We applied the method to data from the Brown region; this gene region is commonly used to infer phylogeny in the genus [33,34] and is used here to better approximate the time period and process involved in the initial divergence of chacma lineages. The input topology required as a prior for the analysis was determined using parsimony and Bayesian inference methods reported in [33]. We tested the hypothesis that lineage divergence was the result of a period of genetic isolation between baboon lineages at the time of an increasingly arid landscape [14]. Demographic parameter priors were input as scalars and we determined present day and ancestral female effective population sizes (*q1* = population 1, *q2* = population 2, and *qA* = ancestral population), migration rates (*m*_*1*_ and *m*_*2*_), and the time to most recent common ancestor (*t*). Initial pilot runs were performed with broad priors and fine-tuned for each parameter. The analysis used the HKY + I + G model [58], selected as the optimal model of nucleotide evolution in MODELTEST v3.6 [60], with an inheritance scalar of 0.25 for mitochondrial DNA [59]. Generation time was set at five years (-u 5) based on average age of first time of reproduction in chacma females [61] and the substitution rate was set at 2% per million years [46]. Using this we estimated a mutation rate of 0.177x10^-4^ per year. MCMC simulations were implemented using five chains with five chain swap attempts per step and a two-step heating increment. Sampling chains were run for 5 million steps (-l 5000000) with a 10% burn-in (-b 500000); under this scenario the lowest effective sample sizes (ESS) for each parameter was at least 500. We executed four independent long runs under identical conditions, with different random number seeds. All four runs produced very similar parameter estimates and 95% highest posterior distributions (HPDs).

### Tests for Late Pleistocene demographic change in chacma baboons

The Middle to Late Pleistocene (~400-12kya) was characterized by a number of glacial-interglacial cycles and attendant changes in precipitation. For example, cycles of increased aridity in the Kalahari region are indicated by repeated periods of dune activation over the last ~100 kyr [62]. Today chacma lineages exhibit a parapatric distribution around the edges of the Kalahari Desert, where they meet along two geographically distinct contact zones (Fig 2). We explored our hypothesis of refugial use and subsequent expansion by testing for evidence of demographic change in the three main mitochondrial clades previously reported [33].

To estimate the time frame for a Bayesian Skyline analysis we first determined the TMRCA for the three clades in BEAST v1.6.1 [63]with a relaxed molecular clock method [64], using the D-loop data collected in this study together with sequences for *P*. *kindae* (GenBank numbers NC020008, JX946202, Zambia) and *P*. *cynacephalus* (GenBank number JX946200, Tanzania). BEAST implements a ‘relaxed phylogenetic’ method in which tree topology and branch lengths are estimated simultaneously from the data [63]. We selected a Yule tree prior (the most suitable prior for trees describing relationships between individuals from different species [63]) and employed a relaxed molecular clock with rates for each branch drawn independently from an uncorrelated lognormal distribution together with the HKY+G substitution model. To calibrate the dating estimate we used fossil calibrated dates obtained from whole mtDNA genome analysis [43]. Values were input as the middle 95% of a normal distribution on each MRCA prior. The split between *ursinus* and the rest of the sample set was set at 2.09 Ma (S.D. 0.3 Ma); and the split between *griseipes*, *kindae* and *cynacephalus* was 1.49 Ma (S.D. 0.2 Ma). We ran two replicates for 10 million generations with tree and parameter sampling every 1000 iterations. The runs were assessed in Tracer v1.4 [63] and the sampling distributions were combined in LogCombiner v1.6.1 with 25% burn-in. The consensus tree was generated in TreeAnnotator v1.6.1 and visualised in FigTree v1.3.1 [65]. We then used D-loop data to estimate Bayesian Skyline Plots of female effective population size change (Ne*f*) in each clade using BEAST v1.6.1 [63]. With standard MCMC sampling BEAST uses a coalescent framework to generate a posterior distribution of effective population size at equally spaced intervals through time [63]. Estimates of population size were grouped into 15 coalescent intervals (m = 15) and the divergence date estimates for the MRCA of all haplotypes within each lineage were used as an upper time limit; a relaxed uncorrelated lognormal molecular clock was employed with mean divergence dates and lognormal standard deviation values to reflect the 95% HPD obtained from the dating estimates for each clade. Mean TMRCA for the *ursinus* clade was set to 1.43 Ma, 1.08 for *griseipes* clade PG1 and 0.96 for *griseipes* clade PG2. We used the HKY + I + G (ModelTest v3.6 [60] and MCMC chains were run for 10 million generations, sampled every 1000 steps. The first 10% of samples were discarded as burn-in; each analysis was run twice and examined for convergence in TRACER v1.4 [63].

Deviations from neutral predictions expected for a constant-sized population were also used to detect past population growth. Using our D-loop data set we performed three tests calculated in DnaSP v5 [66] that consider nucleotide change relative to neutral expectations: Tajima’s *D* [67], R^2^ [68] and Fu’s *F*s [69]. Tajima’s *D* and R^2^ uses the allele frequency spectrum of mutations, while Fu’s *F*s is calculated from haplotype distribution data; for historically stable populations values are expected to be close to zero, while significantly negative *D* and *F*s values suggest population growth. The significance of these tests was evaluated by comparing the observed value with a null distribution simulated under constant population size, generated by 10000 replicates.

### Phylogeographic structure in chacma baboons

To explore our central tenet that chacma baboon populations were repeatedly restricted to refugia during periods of regional climate cycling through the late Pleistocene, we analysed the spatial distribution of genetic variation and inferred more recent demographic change in the PU and PG clades using data from the mitochondrial D-loop. We first used haplotype network analysis to construct intraspecific genealogies for chacma baboons using NeighborNet splits [70] in SPLITSTREE v4.8 [71]. Where reticulation occurs, networks are more sensitive than tree-based criteria to explore fine-scale population structure within lineages [60]. An unrooted NeighborNet network was constructed using an alignment of unique chacma sequences and rooted with D-loop sequences from *P*. *cynacephalus* and *P*. *kindae* (as before); the analysis used uncorrected ‘*p*’ distances and clade structure was tested with 1000 bootstrap replicates. A number of mtDNA phylogenies of *Papio* support the phylogenetic placement of *ursinus* as sister to a clade comprising *griseipes*, *P*. *kindae* and *P*. *cynacephalus*. These two species are distributed across south-central and east Africa, with *P*. *kindae* present in the Miombo woodlands of Angola, the Democratic Republic of Congo, Zambia and possibly western Tanzania; while *P*. *cynacephalus* inhabits savannas and light forest habitats in Somalia, Kenya and Tanzania and southwards to the Zambezi Valley into Zambia, Zimbabwe and across south-central Africa to Angola.

To determine whether chacma clades identified in the network analysis were geographically structured we used a Bayesian assignment approach to test for the presence of distinct evolutionary clusters *K* as defined by their spatial proximity in BAPS v5.2 [72]. The analysis was performed at the individual level using GPS co-ordinates for sampling localities and tests for an upper bound for *K* indicated that the best partition for our dataset was *K* = 5 (2 < *K* < 10; based on the log marginal likelihood values for each partition and the associated posterior probability distributions for each *K* value; S1 Fig). An admixture analysis was then used to estimate the relationships among clusters using a neighbour joining tree based on Nei’s genetic distance. The final partition was visualized using Voronoi tessellation in BAPS.

The Bayesian assignment analysis suggested that chacma baboons comprise spatially distinct evolutionary clusters congruent with the clade topology of the networks. We therefore estimated a number of diversity measures and quantified genetic structure for these clades in Arlequin v3.1 [73]. Estimates of genetic diversity included the number of unique haplotypes (h), haplotype diversity (Hd), nucleotide diversity (π) and the number of segregating sites (S) [74]. The degree to which genetic variation is distributed hierarchically across the landscape was tested using an analysis of molecular variance (AMOVA) [75]. Bootstrapped permutations of the data were used to test how total genetic variance was partitioned into covariance components based on our user-defined clusters.

To further explore the spatial distribution of genetic variation in PU and PG and to identify possible areas that may have acted as barriers to gene flow we used landscape shape interpolation together with Monmonier’s algorithm [76] implemented in Alleles In Space (AIS; [77]. Interpolation routines in AIS were used to construct a 3D surface plot where the x- and y-axes correspond to latitude and longitude coordinates and the z-axis represents genetic distance at a point on the landscape. The analysis first constructs a connectivity network on Delauney triangulation of the spatial co-ordinates for all the locations sampled. Genetic distances between pairs of geographic localities are then calculated and plotted at the midpoint between each location [77,78] and interpolated to create surface peaks that represent areas of large genetic distance between nearest neighbours, together with troughs reflecting areas of low genetic distance. Here, the calculation of the final surface shape is based on the default ‘midpoint of edges’ derived from the Delaunay triangulation and log transformed distance-corrected genetic distances [77]. Monmonier’s algorithm was then used to identify areas of low gene flow across the landscape surface plot.

## Results & Discussion

In concert with glacial expansions through the Pleistocene that caused aridity the vegetated habitats of southern Africa have been repeatedly fragmented [30]. Patterns of diversification in many lineages suggest significant demographic changes that correspond with these cycles, closely approximating proposed habitat and landscape barriers to gene flow, with diverse taxa retreating into refugia until climate amelioration. Most models of climate-driven diversification in southern Africa invoke the shifting dynamics of grasslands, forests and savannas as the dominant cause of phylogeographic structuring. Here we propose and discuss a significant role for an expanded arid landscape in promoting diversification in a highly adaptable primate, adding to our current understanding of the role of landscape change in regional biodiversity dynamics.

### Evidence for lineage isolation during a period of intense aridity

**Fig 3:**
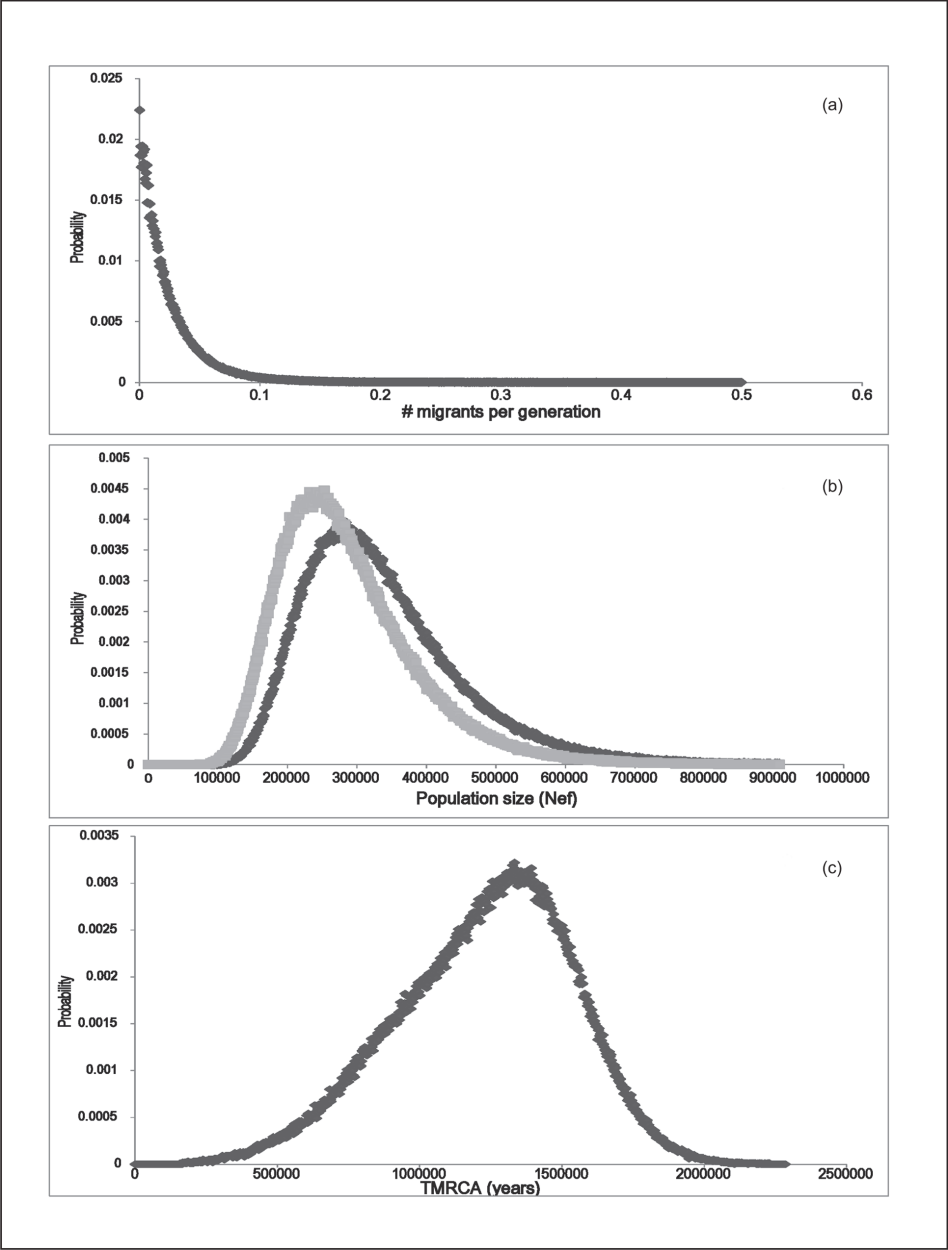
Bayesian posterior probability distributions for tests of lineage isolation among chacma baboon clades. (a) Probability distribution of migration rate *m1* PG (*Papio griseipes*) into PU (*Papio ursinus*). A plot of *m2* PU into PG is equivocal and not shown here. (b) Estimates of Ne*f* for the two major clades: PG (dark grey, Ne*f* = 317000; 95% HPD 172000–608000); PU (light grey, Ne*f* = 261000; 95% HPD 143–428000). (c) Estimate of time to most recent common ancestor (TMRCA) for both lineages (*t* = 1.39Ma; 95% HPD 0.43–2.48Ma).

Coalescent analysis supports a period of isolation with minimal (if any) migration between the two chacma lineages following their divergence during the Pleistocene. Migration probabilities from PU into PG and vice versa both have their highest posterior probability at zero migrants per generation (Fig 3A). Under our alternative models, either *with* migration or *without* migration, we consistently recovered the highest posterior probabilities for migration at zero. Because of problems with achieving full convergence on the parameter of ‘ancestral population size’ under a model with migration, we also estimated Ne*f* (female effective population size) and *t* (TMRCA) without migration. Ne*f* estimated without migration was 261000 (95% HPD 143000–428000) for PU and 317000 (95% HPD 172000–608000) for PG (Fig 3B). Irrespective of the model used, our analyses support previously reported findings [32] of divergence some time during the Mid-Pleistocene (t = 1.39Ma; 95% HPD 0.43–2.48Ma; Fig 3C), when global cooling affected species distribution and diversification patterns worldwide.

A notable gap stands out across the widespread distribution of chacma baboons across southern Africa; their absence from the central Kalahari Desert, located within the southern portion of the much larger 2.5 million km^2^ sand sea. Along with other depositional coastal landforms in southern Africa, this desert formed during the last 3Myr [27,79,79,80]. The Kalahari formation dominates the regional plateau of the south-central African hinterland, and the vaster portion of its aeolian sediments are today stabilized by savanna woodland vegetation [27], but it becomes progressively more arid to the south-west, with exposed dunes and sparse vegetation in the Kgalagadi region of southwest Botswana and northwest South Africa [81]. Geomorphological evidence strongly supports a period of intense aridification in southern Africa from ~2.5–1.7Ma; during this period transverse dunes were reworked and repositioned across the abandoned floor of Palaeo-lake Makgadikgadi [79]. While arid conditions alone do not generally limit the presence of baboons in southern Africa, protracted periods during glacial cycles would have pushed back the zone of accessible surface water in areas surrounding the Kalahari and which arguably would have certainly excluded baboons. For example, the well-studied baboon troops of Tsoabis Leopard Park [82] and the Kuiseb Canyon in Namibia [83] are able to cope with extreme water stress [17] but are nevertheless dependent on regular access to perennial water sources; hence both populations are restricted to the riparian margin in the Namib Desert. The lack of surface water, together with a dearth of suitable sleeping sites, significantly restricts the occurrence of baboons in the Kalahari today, and the past expansion of this desert during Pleistocene glacial likely played an important role in controlling the distribution of *Papio*. We propose that this arid expansion was a primary driver of diversification of the chacma lineage into two clades that today encircle the south-central Kalahari in both the north-east and south-west. Baboons are not the only arid-tolerant mammals to have experienced significant vicariance during this period. Even the hardy African warthog *Phacochoerus spp*. which is capable of surviving in extremely arid conditions (pertinently the historically extinct southern lineage of the desert warthog *P*. *aethiopicus*) experienced genetic isolation during periods of intense aridification in the Pleistocene [11].

### Tests for Late Pleistocene demographic change in chacma lineages

**Fig 4:**
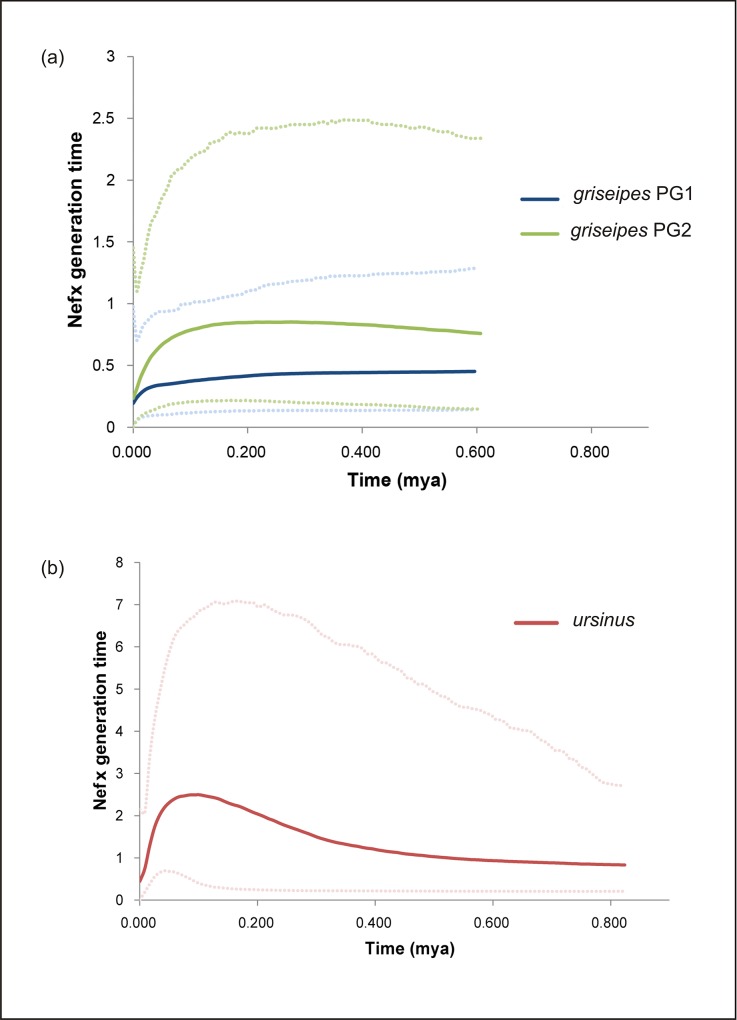
Bayesian Skyline Plots displaying changes in female effective population size (Ne*f*) through time in chacma baboons. Present day is on the left of the x-axis; 95% C.I. are indicated by the dashed lines. Increasing Ne*f* is observed in both PG2 and PU over the mid to late-Pleistocene, while all three clades reveal declining Ne*f* through the Holocene to the present day.

The recent demographic history of chacma mtDNA lineages were reconstructed using Bayesian skyline plots, where MCMC integration under a coalescent model allows us to explore changes in Ne*f* through time. The resulting plots reveal an extended period of increasing population size for both the PU and the PG2 lineage (Fig 4). Disparities in the timing and trends of population size change between the plots suggest independent histories for each lineage. Ne*f* of the PU lineage is substantially larger than either that of the north eastern (PG2) or north western (PG1) *griseipes* clades. Both PU and PG2 indicate a period of population growth from the mid-Pleistocene. In PU we see population size increase beginning at ~800ka. This trend continues beyond ~460ka, at this point the relative rate appears to increase. This is then followed by a slow down at ~160ka. A period of decline in Ne*f* is suggested at ~90ka, which then slows down in the early Holocene. Within *griseipes* the BSPs indicated distinct differences in the demographic histories and female effective population sizes for PG1 and PG2. In PG2, the larger of the two *griseipes* lineages, population size slowly increases from the mid-Pleistocene, expanding from ~600ka to ~180ka. At this point while growth in PU is beginning to plateau, PG2 begins a period of slow decline into the Holocene. Over the same time period PG1 experiences a slow decline from ~600ka, increasing in the rate of decline into the Holocene. Tests for neutrality using the D-loop data set indicate that regional clades in both PU and PG clade are out of mutation-drift equilibrium, which may be explained by regional (and/or possibly local) demographic shifts such as population expansion. Significant values for Tajima’s *D* and R^2^ were found in PU clades 2 and 3 (Tajima’s *D* p≤0.05, R^2^ p = 0.01 for all clades in PU), while overall both PU and PG clades supported significant R^2^ values (p = 0.05).

The historical population trends inferred by these analyses correspond closely to the dynamic regional climate trends of southern Africa since the mid to late Pleistocene. The persistence of of baboons, indicated by molecular and fossil evidence, after 1.5Ma coincides with the Plio-Pleistocene domination of arid adapted species in the biodiversity of Africa [31]; this is a very interesting finding and may reflect the ability of baboons to endure a broad range of ecological conditions while less tolerant species declined and/or went extinct ([31] and references therein, [21]). For example, the latest appraisal of admittedly controversial OSL dating of the Kalahari and Namib dune systems suggests repeated periods of dune reactivation in the Late Pleistocene (from 190 ka) through to the present day [84]; baboons have clearly persisted through these periods of expanding desert conditions, and can today be found along the permanent waterways occurring in these landscapes e.g. the Okavango Delta, Botswana and the Kuiseb River in Namibia which flows from the Khomas Highlands west to the Atlantic Ocean in Namibia. Additionally, the overall pattern of increasing Ne*f* observed in both PU and PG2 from the mid to terminal Pleistocene may be the result of a number of prolonged periods of interglacial warming; this pattern is evident in a number of African taxa e.g. Cape buffalo *Syncerus caffer*, [85] which increased during a period of forest expansion across the eastern Great Escarpment of southern Africa [86] during Marine Isotope Stage 11 (MIS 11; 427–364ka; [87,88]. During MIS 6, 186-130ka [89] this climatic trend then reversed and has been flagged as archeologically significant [90,91], particularly with the first appearance of Late Middle Stone Age cultures in central Africa and anatomic and behavioural modernity appearing to take root [25,92]. Some authorities suggest these adaptations are linked to repeated periods of possible isolation [93] and likely affected the mammalian fauna of the region as well, leading to some degree of repeated fragmentation [33].

At the onset of the Holocene, a period of distinct ecological transitions occurred. High-resolution stable carbon and nitrogen isotope records suggest a wetter early Holocene and a later dry period [94], after 3500 BP. These wetter phases correlate with forest expansions [94] that possibly fragmented many habitats, and the lower Ne*f* observed in all three chacma clades may reflect a period of lineage divergence. This process needs further exploration with finer-scale sampling of baboons across the landscapes of southern Africa.

### Phylogeographic structure in chacma baboons—lineage diversification over the Late Pleistocene

**Fig 5:**
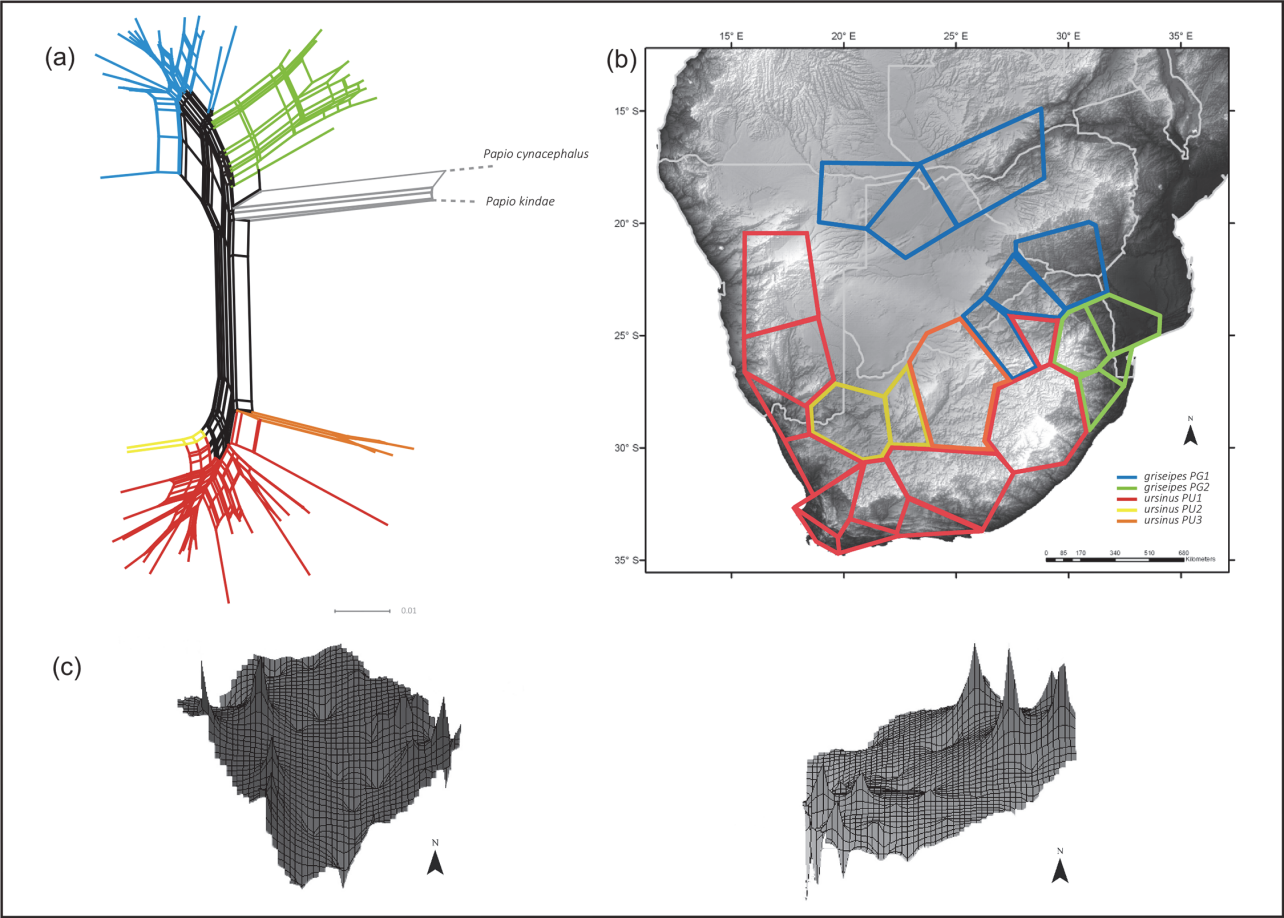
Spatial genetic diversity and population structure in chacma baboons across southern Africa. (a) Rooted NeighbourNet network for *ursinus* and *griseipes* chacma lineages together with *Papio cynacephalus* and *P*. *kindae*. Numerous recent studies based on mtDNA reveal *griseipes* as sister to these two more northerly species. (b) Bayesian analysis of spatial genetic structure reveals distinct geographic clustering in both lineages. PG1—blue; PG2—green; PU1—red; PU2—yellow; PU3—orange. (c) Landscape plots of genetic diversity across the sampling distributions of the PG (left) and PU (right) clades in southern Africa; the plots are oriented north-south.

Splitstree networks for the PG and PU D-loop lineages (Fig 5A) indicate two distinct clades in PG and three in PU. Estimates of genetic variation across these clades are reported in Table 1. Spatial analysis using BAPS (Fig 5B) together with AMOVA (Table 2) both support clear geographic structuring in chacma baboons and provide good support for strong regional phylogeographic structure. The AMOVA analysis revealed significant population genetic differentiation among clades, with ~45% of the variation distributed among ‘groups’ (PU and PG), and ~31% among ‘populations’ i.e. the five clades. Given the effects of climate cycling on vegetation in this region it is most likely that differentiation among chacma lineages occurred via drift, most likely via isolation and changing population sizes. Landscape visualization of genetic distance (reflecting underlying genetic diversity; Table 1) generated in Alleles in Space (Fig 5C) also suggest diverse histories in chacma baboons. In the PG lineage, high genetic distance (diversity) occurs in the west and east, associated with the distribution of the PG1 and 2 clades respectively, while in the PU lineage, the highest genetic diversity occurs in the PU1 and 2 clades, which together with PG1 border the Kalahari Desert. When considered together, these plots are most likely consistent with a model of contraction into vegetated refugia and subsequent recolonization of the surrounding landscape.

**Table 1:** Diversity descriptors and estimators of demographic change for chacma baboons sampled across southern Africa, based on mtDNA D-loop sequence data.

**Taxon**	Clade	Diversity Indices			Neutrality Tests					
		h	Hd	π (±S.E.)	Tajima’s *D*	*p*-value	Fu’s F_*S*_	*p*-value	R^2^	*p*-value
***griseipes* (PG)**	*griseipes* clade (n = 72)	31	0.96	0.11 (±0.05)	-0.22	p = 0.48	-1.72	p = 0.34	0.07	**p = 0.05**
	Clade PG1 (n = 44)	23	0.92	0.06 (±0.03)	-1.19	p = 0.10	-3.11	p = 0.17	0.08	**p = 0.05**
	Clade PG2 (n = 28)	8	0.87	0.05 (±0.03)	1.43	p = 0.93	3.76	p = 0.92	0.14	p = 0.7
***ursinus* (PU)**	*ursinus* clade(n = 57)	30	0.96	0.13 (±0.06)	-0.67	p = 0.27	-0.28	p = 0.51	0.08	**p = 0.05**
	Clade PU1 (n = 38)	21	0.96	0.09 (±0.05)	-0.42	p = 0.38	-0.90	p = 0.41	0.16	**p = 0.01**
	Clade PU2 (n = 8)	2	0.95	0.03 (±0.01)	-1.82	**p = 0.004**	6.46	p = 0.95	0.16	**p = 0.01**
	Clade PU3 (n = 15)	6	0.76	0.09 (±0.05)	-1.52	**p = 0.05**	6.32	p = 0.98	0.16	**p = 0.01**

PU—*Papio ursinus* clade; PG—*Papio griseipes* clade.

**Table 2:** Analysis of molecular variation in southern African chacma baboons based on mtDNA D-loop sequence data.

	*Among group*	*Among populations within groups (% variation)*	*Within populations(% variation)*
Group 1 *ursinus* v Group 2 *griseipes*	45.05	31.16	23.79
Group 1(PU1,2 & 3)		50.19	49.91
Group 2(PG1 & 2)		62.73	37.26

Phylogeographic studies of numerous southern African species reveal patterns of genetic structuring suggestive of repeated use of glacial refugia and subsequent expansion and fragmentation of lineages [95–103]. The PUD3 clade extends from central Namibia south to the Cape Peninsula, continuing northeast to the Drakensberg-Maluti mountain range. Clade structure in Smith‘s red rock rabbit *Pronolagus rupestris* shares similarities with the distribution of PU3, where rock rabbit populations are confined to the mountain ranges and abutting regions of the Great Escarpment of South Africa [104]; the rock rabbit clades represent relict montane populations that subsequently seeded later expansions [104]. We propose the PU3 clade of *Papio* may have experienced a congruent history to that of *Pronolagus rupestris*, and represent the dispersal of a relict population that took refuge in the Mediterranean-type habitat of the south-western Cape, later expanding out of this glacial refuge along the coastal Cape Fold Mountains towards the north and east. A similar pattern is reported in rock hyrax *Procavia capensis* with the distribution of a south-eastern clade similarly confined along the Great Escarpment mountain range [105]. Both of these studies also recovered additional clades associated with the high-lying Kuruman Hills (~1467 m above sea level near the Augrabies Falls in South Africa [105,106], an area suggested to be a refugium and subsequent source for recolonization across the marginal habitat of the Northern Cape plains [106]. The distribution of these high-lying clades closely approximates that of PU2, and this may represent a similar dispersal event away from the expanding PU1 during favourable conditions, and later surviving as an outlier in this montane refuge during an ensuing glacial. Similar processes may be invoked to explain the divergence of PU3. Nonetheless, the ranges of PU2 and PU3 are both bounded by the Kalahari in the north, suggesting this landform has been a constant barrier to successful dispersal and gene flow.

Given our lack of information from the regions directly to their north, reconstructing the possible history of the *griseipes* clades is more complicated. Nonetheless, the high diversity in the east along the Kalahari (PG1) mirrors that characterizing the PU2 and PU3 clades and it’s likely that this arid interior also played an important role in structuring diversity. The PG2 clade is centered on a region bounded to its west by the Drakensberg-Maluti Mountains and with dense coastal forests in the north. The high diversity seen at the eastern most boundary of this clade might reflect a comparable colonization event from montane refugia in the Drakensberg analogous to the Kuruman Hills (above). However, interpretation of diversity in both of the PG clades demands caution, given the likelihood of gene flow from further north. It is important to note here that the Early Pleistocene pulse of diversification in *Papio* that gave rise to the north-eastern PG clade also founded the *kindae* and *cynocephalus* lineages [43]. Thereafter, PU persisted as a single lineage isolated to the southwest and east of these species. Further understanding of the distribution, diversity and history of these clades requires greater geographic sampling and the addition of nuclear markers to this dataset.

## Conclusions and Taxonomic Implications

Together with recent studies [33,34,107] the results reported here provide strong evidence that the chacma baboons of southern Africa do not comprise a single monophyletic taxon. Indeed, the two lineages discussed here emerge early in the evolutionary history (ca. 1.9–1.6Ma) of *Papio*, and have persisted in geographical and genetic isolation since the Early Pleistocene. Furthermore, they each continued to evolve along distinct evolutionary trajectories into the Holocene. Phylogeographic reconstructions indicate that the south-western *ursinus* group contracted into at least three geographically isolated refugia, likely as a result of aridification, and this metapopulation has only increased its range quite recently as climate ameliorated. In contrast, the *griseipes* baboons in the north-east likely had a more complicated history, originating from a lineage that later diversified into ‘*griseipes’*, *P*. *kindae* and *P*. *cynocephalus* [43]. Structuring of diversity within this more northerly taxon has also likely been affected by some degree of ongoing genetic exchange with these more northerly baboons. Given these deep and notably different histories, combined with morphological evidence that has long pointed to their taxonomic distinctiveness [37,40,44], we argue these lineages are as distinctly differentiated as other recognized baboon species. Indeed, behavioural evidence, although certainly not continuous across the range, also suggests very different adaptive repertoires in different regions (e.g. seafood foraging in low nutrient environments [108] and persistence into the snow-line of the high alpine Drakensberg Mountains [83,109]. Within the framework of the Evolutionary Species Concept [110, 111] we suggest that formal separation at the species level into *Papio ursinus* (south-west) and *Papio griseipes* (north-east) might be warranted. However, further studies into the geographic distribution and genetic make-up of these taxa, including foci on nuclear gene regions [107] together with mitogenomic approaches [112], and the position and nature of secondary contact zones, are needed to confirm this. Regardless of taxonomic status, these studies will help further our understanding of variation in the notably diverse southern African baboons.

## Supporting Information

S1 Fig Changes in log-likelihood estimated in BAPS for K = 2–5 [72]. Individual level mixture analysis supports K = 5 as the most likely number of groups in the optimal partition based on the log (ml) values for the size of the 10 best visited partitions.
(TIF)

S1 Table Sampling location details and *Papio* clade identity for individuals used in this study. Map ID numbers correspond to Fig 1. Biome designations for sampling locations follow [113–115].
(DOCX)
