# Supplementary figures and images for: Pleistocene Aridification Cycles Shaped the Contemporary Genetic Architecture of Southern African Baboons
Source: PLoS One. 2015 May 13;10(5):e0123207. doi: 10.1371/journal.pone.0123207 (PMC4430493; doi:10.1371/journal.pone.0123207)

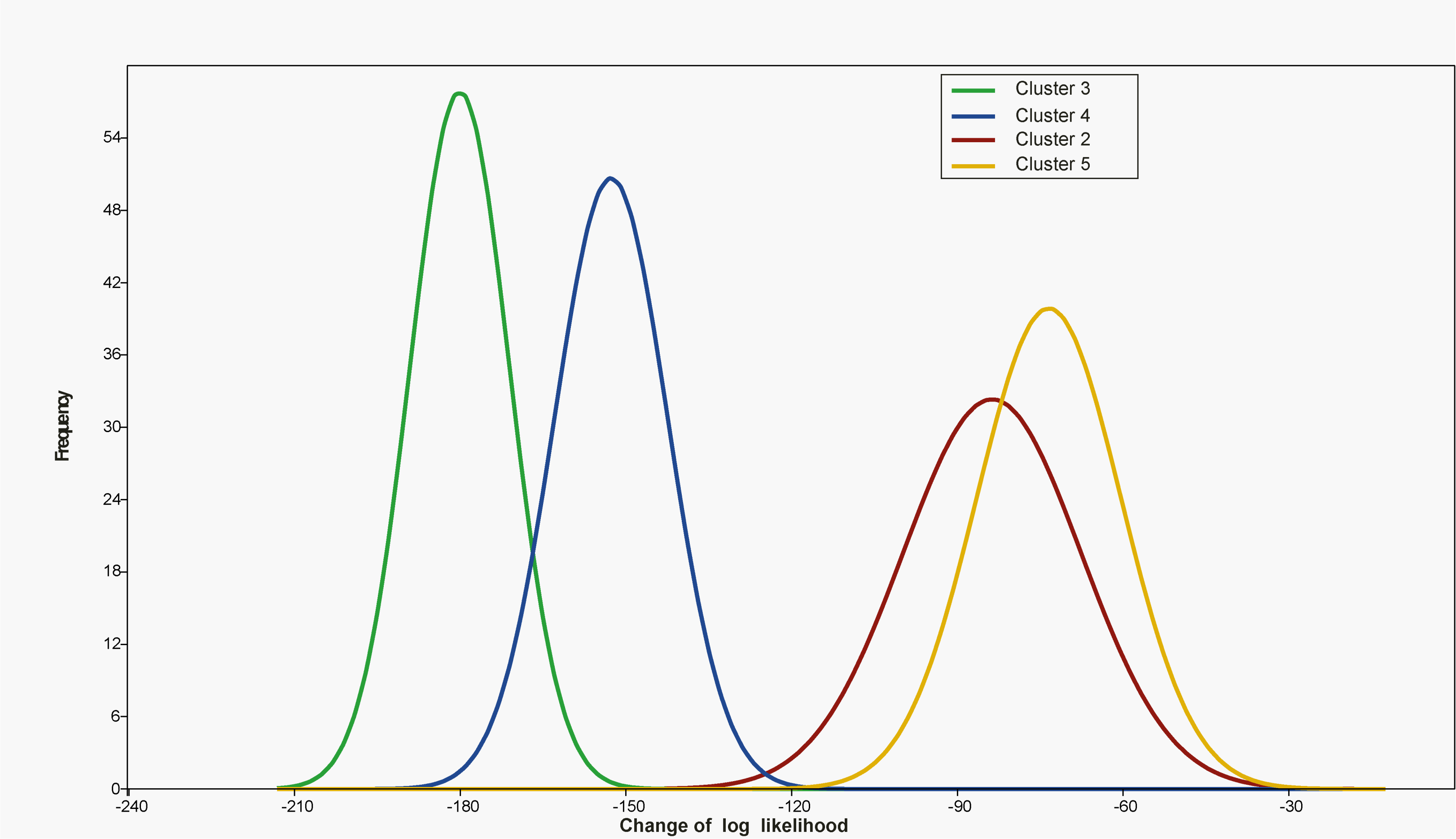

Supplement: S1 Fig — Individual level mixture analysis supports K = 5 as the most likely number of groups in the optimal partition based on the log (ml) values for the size of the 10 best visited partitions. (TIF) [file pone.0123207.s001.tif]
