# Supplementary material for: Pleistocene Aridification Cycles Shaped the Contemporary Genetic Architecture of Southern African Baboons
Source: PLoS One. 2015 May 13;10(5):e0123207. doi: 10.1371/journal.pone.0123207 (PMC4430493; doi:10.1371/journal.pone.0123207)
Supplement: S1 Table — Map ID numbers correspond to Fig 1. Biome designations for sampling locations follow [113–115]. (DOCX) [file pone.0123207.s002.docx]

**S1 Table. Sampling location details and *Papio* clade identity for individuals used in this study.** Map ID numbers correspond to Figure 1. Biome designations for sampling locations follow [113-115].

| **Map ID** | **Location** | **Country** | **GPS Co-ordinates (decimal degrees)** | | **Biome** | ***Papio* Clade** |
| --- | --- | --- | --- | --- | --- | --- |
| 1 | Rundu (n=7) | Namibia | 21.6043 | -18.1525 | Riparian Woodland | *griseipes* |
| 2 | Okahandja (n=2) | Namibia | 16.9166 | -21.9833 | Thornbush Savannah | *ursinus* |
| 3 | Swakopmund (n=1) | Namibia | 14.5333 | -22.6833 | Desert | *ursinus* |
| 4 | Kheetmanshoop (n=1) | Namibia | 18.1466 | -26.5762 | Nama-Karoo | *ursinus* |
| 5 | Kuboes (n=6) | South Africa | 16.9967 | -28.4411 | Nama-Karoo | *ursinus* |
| 6 | Augrabies National Park (n=7) | South Africa | 20.3201 | -28.5470 | Nama-Karoo | *ursinus/ griseipes* |
| 7 | Vanrhynsdorp (n=1) | South Africa | 18.7541 | -31.6004 | Nama-Karoo | *ursinus* |
| 8 | Dwarsrivier (n=2) | South Africa | 29.7500 | -23.4500 | Mountain Fynbos | *ursinus* |
| 9 | Elandsbaai (n=2) | South Africa | 19.4509 | -32.8005 | Coastal Fynbos | *ursinus* |
| 10 | Table Mountain National Park (n=5) | South Africa | 18.4071 | -34.1576 | Fynbos | *ursinus* |
| 11 | Calitzdorp (n=7) | South Africa | 21.4706 | -34.0243 | Fynbos | *ursinus* |
| 12 | Die Hel (n=3) | South Africa | 21.7157 | -33.3439 | Succulent Karoo | *ursinus* |
| 13 | Nature’s Valley (n=10) | South Africa | 23.6196 | -33.9493 | Forest | *ursinus* |
| 14 | Nieu Bethesda (n=9) | South Africa | 24.5548 | -31.8664 | Nama-Karoo | *ursinus* |
| 15 | Kimberley & Barkly West (n=11) | South Africa | 24.7666 | -28.7333 | Savannah | *ursinus/ griseipes* |
| 16 | Golden Gate National Park (n=6) | South Africa | 28.9619 | -28.5385 | Grassland | *ursinus/ griseipes* |
| 17 | Royal Natal Park (n=3) | South Africa | 28.9619 | -28.5385 | Grassland | *griseipes* |
| 18 | Hluhluwe-Imfolozi National Park (n=6) | South Africa | 32.2694 | -28.0087 | Grassland | *ursinus/ griseipes* |
| 19 | Kosi Bay (n=1) | South Africa | 32.8673 | -26.9062 | Grassland | *griseipes* |
| 20 | Kruger National Park (n=5) | South Africa | 31.6383 | -24.4861 | Savannah | *griseipes* |
| 21 | Blyde River Canyon (n=11) | South Africa | 30.8465 | -24.8612 | Grassland | *griseipes* |
| 22 | Loskop Dam Nature Reserve (n=7) | South Africa | 28.2108 | -24.4351 | Savannah | *ursinus/ griseipes* |
| 23 | Rustenburg (n=2) | South Africa | 27.3173 | -24.9472 | Savannah | *griseipes* |
| 24 | Lapalala Nature Reserve (n=11) | South Africa | 28.2594 | -23.8424 | Savannah | *griseipes* |
| 25 | Musina (n=5) | South Africa | 30.0398 | -22.3495 | Savannah | *griseipes* |
| 26 | Matobo Hills (n=1) | Zimbabwe | 28.4778 | -20.4088 | Savannah Mosaic | *griseipes* |
| 27 | Chizarira (n=1) | Zimbabwe | 27.8833 | -17.7833 | Semi-arid Savannah | *griseipes* |
| 28 | Choma (n=3) | Zambia | 25.8553 | -17.8535 | Miombo Woodland | *griseipes* |
| 29 | Moremi Game Reserve  (n=10) | Botswana | 23.1035 | -19.4648 | Savannah Wetland Mosaic | *griseipes* |
